# Deprescribing Decisions in Swiss Primary Care: Low Concordance Between General Practitioners and Older Adults

**DOI:** 10.1007/s11606-025-10081-z

**Published:** 2026-01-14

**Authors:** Kristie Rebecca Weir, Renata Vidonscky Lüthold, Zsofia Rozsnyai, Sven Streit, Katharina Tabea Jungo

**Affiliations:** 1 Institute of Primary Health Care (BIHAM), University of Bern, Bern, Switzerland; 2 Sydney School of Public Health, Faculty of Medicine and Health, University of Sydney, Sydney, Australia; 3 Graduate School for Health Sciences, University of Bern, Bern, Switzerland; 4 Division of Pharmacoepidemiology and Pharmacoeconomics and Center for Healthcare Delivery Sciences (C4HDS), Department of Medicine, Brigham and Women’s Hospital and Harvard Medical School, Boston, MA USA

**Keywords:** older patients, cross-sectional survey, attitudes, medications

## Abstract

**Background:**

Deprescribing reduces potentially harmful or unnecessary medications in older adults. Successful implementation requires understanding both patient and provider perspectives, although few studies have compared their preferences.

**Objective:**

To examine general practitioner (GP) and patient preferences for deprescribing and medication-related decision-making by investigating factors associated with preference concordance and the relationship with patient-provider trust.

**Design:**

Cross-sectional survey study conducted in primary care settings within the German-speaking region of Switzerland.

**Participants:**

Sixty-five patients aged ≥ 65 years taking ≥ 5 medications and their 10 GPs completed questionnaires.

**Main Measures:**

Patient and GP preferences for deprescribing medications were assessed through two questions; for patients: “Thinking about your current medication list, are there any medications that you would like to stop taking or reduce the dose of?” and for GPs: “Would you stop or reduce the dose of any of the medications that the patient is currently taking?” We assessed concordance between GPs’ and patients’ deprescribing preferences and analyzed associations between trust and deprescribing preferences using univariate Generalized Estimating Equations with Poisson distribution and finite-sample correction, accounting for clustering at the GP level.

**Key Results:**

Similar proportions of patients (38%) and GPs (35%) wanted to deprescribe at least one medication, but only eight GP-patient dyads wanted to deprescribe, with just one dyad selecting the same medication. The most frequently identified medications to deprescribe were dietary supplements (*GPs* = 11/42, 26%; *patients* = 4/35, 11%), cardiovascular system medications (*GPs* = 9/42, 21%; *patients* = 15/35, 43%), and nervous system medications (*GPs* = 8/42, 19%; *patients* = 9/35, 26%). GPs’ primary reason for not deprescribing was believing patients wanted to continue their medications (83%), while patients believed doctors only prescribe necessary ones (38%).

**Conclusions:**

Despite similar interest in deprescribing, GPs and patients rarely selected the same medications to stop. These findings suggest that clear discussions about medication necessity and preferences could improve deprescribing decisions.

**Supplementary information:**

The online version contains supplementary material available at 10.1007/s11606-025-10081-z.

## INTRODUCTION

Polypharmacy is a global health issue, with many older adults prescribed potentially harmful or unnecessary medications^1^. Depending on the individual’s health, goals, and preferences, the harms of these medications may outweigh their benefits, making deprescribing an appropriate option^2^. However, implementing deprescribing effectively requires collaboration between healthcare providers and patients, and an understanding of the relationship between them^3^.

General practitioners (GPs) and older adults influence deprescribing decisions in connected ways. Both GPs and patients have expressed hesitation to discontinue long-term medications, citing concerns about potential negative consequences or perceived benefits of the medication^4,5^. The beliefs and expectations of both parties significantly impact medication decisions, with an Australian study of 22 GPs and 336 patients finding that patients were ten times more likely to be prescribed a medication if the GP perceived they expected it, and nearly three times more likely if patients themselves expected to receive a prescription^6^. While patients’ concerns about side effects, medication burden, or changing health goals can prompt conversations about deprescribing^7–9^, GPs’ direct recommendations significantly increase patients’ willingness to consider stopping a medication^10^.

Trust in primary care is often viewed through the lens of patients’ trust in their GP^11^, yet feeling trusted and respected by doctors can powerfully shape the patient experience^12,13^. Older adults generally report high trust in their GP’s competence^14^which is reinforced when patients feel believed and taken seriously^13^. While trust plays a key role in medicine-related decision-making^15,16^, its specific influence on deprescribing is not yet fully understood.

Although patients’ attitudes towards medications influence their willingness to deprescribe, current research in this area has two key limitations. First, studies usually focus on attitudes towards medications in general, rather than the specific medications the patient is taking^17^. Second, most studies examine the perspectives of healthcare professionals^18^ or patients^19^ in isolation. Research comparing how both groups view individual medications and their preferences for deprescribing outside of interventional settings is lacking. This study addresses these gaps by examining and comparing GPs’ and patients’ medication-related decision-making as well as their preferences for deprescribing. We also explored the association between deprescribing preferences and patient-provider trust.

## METHOD

This manuscript reports findings from a preplanned Swiss study of a larger multi-country project examining older adults’ attitudes towards deprescribing^20^^,21^. This cross-sectional study was conducted from May 2022 to December 2023, with older adults and GPs recruited in the German-speaking part of Switzerland.

Switzerland’s healthcare system is highly decentralized. As cantons are responsible for key services, notable regional differences exist^22^, for example, in some cantons GPs can dispense medications directly to patients^23^.

The study was approved by the competent local ethics committee in Switzerland (*Kantonale Ethikkommission Bern*) (Project-ID 2022–00035). The survey was completed by GPs and patients either online using REDCap or on paper. Full details are available in the published protocol^20^^,21^.

### Study Design and Sample

Recruitment strategies included advertising through newsletters, GP conferences, and mailing lists; contacting GPs who had previously consented to be contacted for future research; and cold emailing or sending letters to GPs using publicly available information. Due to difficulties in recruitment, we introduced a lottery incentive (offering three of all respondents (87) the chance to win a voucher of 100CHF), although this did not improve participation. GPs who recruited a minimum of five participants were offered 200CHF to recruit an additional five to ten participants; yet only two GPs accepted this offer. GPs were asked to recruit patients aged 65 years and over, taking 5 or more medications regularly. Patients were ineligible if they could not give informed consent or if they resided outside of Switzerland. GPs were instructed to consecutively recruit eligible patients to have a representative sample of patients. GPs were asked to screen their consultations of the day and flag eligible patients, who were then invited to participate in the study during the consultation. Participating GPs were from the following cantons: Aargau, Bern, Luzern, St. Gallen, and Zurich.

### Outcome Measures

Our primary outcomes were patients’ and GPs’ interest in deprescribing.

*Patients’ interest in deprescribing*: Patients were asked, “Thinking about your current medication list, are there any medications that you would like to stop taking or reduce the dose of?” (Yes/No). Those who responded “yes” could list up to four medications. Patients who answered “yes” or “no” selected reasons from a pre-determined list, adapted from Vordenberg et al.^24^ including options such as cost, convenience, and side effects (Appendix patient survey Q25–26).

*GPs’ interest in deprescribing*: GPs were asked, “Would you stop or reduce the dose of any of the medications that the patient is currently taking?” (Yes/No). Those who responded “yes” could identify any number of specific medications from the patient’s list.

### Variables

Table 1 summarizes the key variables collected from patients and GPs, along with the corresponding survey questions (see Appendix: patient and GP surveys). Data were pseudonymized to match GPs and patients for the analysis.

**Table 1:** Summary of Patient and GP Measures

Measure	Description	Source	Question in survey
Patients			
Trust in GP	Five questions (scores ranged = 5–25), dichotomised into higher trust (≥ median) and lower trust (< median), given the skewed distribution	Abbreviated Wake Forest Trust Scale^25^	Q31–Q35
Attitudes towards medicines and decision-making preferences	Three questions about patients’ beliefs about medication importance, learning style, and decision-making preferences. For each question, patients selected one response that best aligned with their attitudes, beliefs, and preferences	Patient Deprescribing Typology^16,26^ ^,27^	Q27–Q30
Satisfaction with medications	Satisfaction with medications was measured using one question (5-point scale from “Strongly disagree” to “Strongly agree”)	Revised Patients’ Attitudes Towards Deprescribing quesitonnaire^28^	Q22
Medications for deprescribing	Patients identified up to four medications based on their memory. Anatomical Therapeutic Chemical codes at the second anatomical level were used to classify medications and group them into specific therapeutic and pharmacological subcategories^1^.	World Health Organization^29^ for classification, using the Anatomical Therapeutic Chemical codes	Q25
Demographics	Country, gender, education, financial status, health literacy, self-rated health, number of medications, amount of support needed for managing their medications, and relationship duration with their GP	Health literacy^30,31^ Self-rated health^32^ Financial status^33^	Q1–Q21 Q23 Q16
GPs			
Trust and engagement	GPs rated their agreement with 4 statements on their perception of patient trust and engagement (5-point scale from “Strongly disagree” to “Strongly agree”)	Developed by the study team	Q16–Q19 (Part 2)
Decision-making	GPs were asked two questions about how they would *usually* and how they would *like* to make decisions about deprescribing with a patient. For each question, GPs selected one response that best aligned with their decision-making	Adapted from Driever et al^34^., who modified the Control Preference Scale^35^	Q20–Q21 (Part 1)
Goal and preference elicitation	Importance: GPs rated how important they believe it is to understand their patients’ goals and preferences about medications (5-point scale from “Not at all important” to “Really important”). Frequency: GPs reported how often they discuss patients’ goals and preferences about medications (5-point scale from “Never” to “Always”)	Adapted from a GP typology about patients’ goals and preferences^36^	Q18–Q19 (Part 1)
Deprescribing and polypharmacy	GPs estimated % of patients with polypharmacy, % eligible for deprescribing, and % they had recommended deprescribing to	Adapted from the Prescribers’ Perceptions of Medication Discontinuation Survey^18^	Q13–Q17 (Part 1)
Medications for deprescribing	GPs could select any number of medications from their patient’s list for deprescribing. (See Patients, Medications for deprescribing)	World Health Organization^29^ for classification, using the Anatomical Therapeutic Chemical codes	Q7–Q8 (Part 2)
Demographics	Gender, age, first language, years of work experience, average consultations per day, practice location and type, and their patients’ number of medications taken	Developed by the study team	Q1–Q12 (Part 1) (Part 2)

^1^Given the lack of consensus on how to classify dietary supplements with ATC codes, they are classified as a separate category

### Statistical Analysis

Descriptive statistics were used to summarize participant characteristics and attitudes, with continuous variables presented as means (SD) or medians (IQR) and categorical variables as counts and percentages. We also described GP and patient characteristics, attitudes, trust, and decision-making preferences by whether GP and patients, respectively, expressed an interest in deprescribing or not.

To assess concordance between GPs’ and patients’ deprescribing choices, we created a variable indicating whether their preferences matched (or not) for each medication. Each medication was classified as (i) Concordant: both GP and patient chose to deprescribe it (yes/no); (ii) Discordant: only one chose to deprescribe it (yes/no). A descriptive analysis was conducted.

Associations between trust and deprescribing preferences were examined using univariate Generalized Estimating Equations (GEE) with a Poisson distribution and log link used to estimate risk ratios, accounting for the correlation of responses within GP clusters^37^ and using robust standard errors. Given the small number of clusters (*n* = 10 GPs), we applied a Kauermann-Carroll finite-sample correction to the standard errors to reduce the risk of Type I error^38^(using the xtgeebcv command in Stata)^39^. We used Poisson regression with a log link rather than logistic regression because the outcome (interest in deprescribing) was common. In this context, risk ratios provide a more interpretable measure of association and are preferred over odds ratios^37^. Missing data was handled using complete case analysis. Statistical analysis was performed using Stata 16.1, with a two-sided *p*-value of ≤ 0.05 considered significant.

## RESULTS

### Characteristics of Participants

Of the patients, 29 (45%) were women and 35 (54%) were men, and 33 (51%) had secondary school as their highest educational level, 40 (62%) were “*extremely/quite a bit confident*” in filling out medical forms (Table 2). Patients were taking an average of 6 regular medications (IQR = 3), most rated their overall health as “*average/good*” (57, 87%), and 33 (51%) had been seeing their GPs for 10 or more years.

**Table 2:** Patients’ and General Practitioners’ Characteristics

Patients’ characteristics (*n* = 65)	*n* (%)
**What is your gender?**	
Female	29 (45%)
Male	35 (54%)
Other	0 (0%)
Missing	1 (2%)
**What is your highest completed education?**	
None	2 (3%)
Primary school	20 (31%)
Secondary school	33 (51%)
Third level education	9 (14%)
Missing	1 (2%)
**How do you make ends meet financially?**	
Without any problems	26 (40%)
Quite easily	31 (48%)
With some difficulty	6 (9%)
With great difficulty	1 (2%)
Missing	1 (2%)
**How confident are you filling out medical forms by yourself?**	
Extremely	12 (19%)
Quite a bit	28 (43%)
Somewhat	16 (25%)
A little bit	7 (11%)
Not at all	2 (3%)
**Where were you born?**	
In the country where I currently live	58 (89%)
In another country	6 (10%)
Missing	1 (2%)
**In general, how would you describe your health today?**	
Excellent	0 (0%)
Very good	2 (3%)
Good	36 (55%)
Average	21 (32%)
Poor	4 (6%)
Missing	2 (3%)
**Overall, I am satisfied with my current medications**	
Strongly disagree	0 (0%)
Disagree	0 (0%)
**Unsure**	5 (8%)
Agree	43 (66%)
Strongly agree	17 (26%)
**How many different kinds of medications do you take regularly, **median IQR	6 (3)
**Do you prepare your medication by yourself?**	
Yes	60 (92%)
No	5 (8%)
Missing	4 (6%)
**How long have you been seeing this GP?**	
0–9 years	30 (46%)
10–19 years	18 (28%)
20–29 years	13 (20%)
30+ years	2 (3%)
Missing	2 (3%)
**GPs’ characteristics** (*n* = 10)	
**Gender**	
Female	4 (40%)
Male	6 (60%)
**Age (years)**, median (IQR)	56 (12)
**First language** (multiple responses possible)	
German/Swiss German	9 (90%)
French	2 (20%)
**Work experience (years)**, median (IQR)	15 (9)
**Practice location**	
In an urban area	4 (40%)
In a suburban area	1 (10%)
In the countryside	5 (50%)
**Type of practice**	
Single practice	2 (20%)
Group practice	8 (80%)
**Average consultations per day**	
≤ 25	9 (90%)
26+	1 (10%)
**Number of regular medications of recruited patients,** median IQR	9 (5)
Missing	1 (2%)
**Deprescribing and polypharmacy**	
**Familiar with the concept of deprescribing?** (yes)	10 (100%)
GP-estimated percentage: Patients in your practice who have polypharmacy (i.e., who regularly take 5 or more medications), median, IQR	35% (30%)
GP-estimated percentage: Patients in your practice who are eligible for stopping or dose reduction, median, IQR	25% (30%)
GP-estimated percentage: For patients taking medications that could potentially be stopped or reduced, what percentage do you estimate you have recommended this to, median, IQR	70% (60%)
Missing	4 (6%)

The percentage of missingness is only shown for variables with missing data

Of the GPs, 4 (40%) were women and 6 (60%) were men (Table 2). They had an average of 15 years of work experience as a GP (IQR = 9), and 80% (*n* = 8) worked in a group practice (minimum of two GPs). All participating GPs (100%) were familiar with the concept of deprescribing prior to the study. GPs estimated that a median of 35% (IQR = 30%) of their patients were taking 5 or more medications and that 25% (IQR = 30%) of their patients were eligible for deprescribing. GPs estimated that they had recommended deprescribing to a median of 70% (IQR = 60%) of those patients.

### Concordance Between GPs’ and Patients’ Preferences Towards Deprescribing

A total of 65 GP-patient dyads were included in the study, with each GP connected to a median of 5 patients (range = 4 to 16 patients). Overall, there were similar proportions of patients who responded “yes” to whether they would like to deprescribe a medication and patients whose GPs responded they would like to deprescribe the patient’s medication (Table 3). Thirty-eight percent (25/65) of patients and 35% (23/65) of GPs expressed a preference to deprescribe at least one of the patient’s medications. Patients identified a median of 1 medication per patient (IQR = 1) and GPs identified a median of 2 medications per patient (IQR = 5). In 24 of the 65 GP-patient dyads, both the GP and patient stated they did not want to stop a medication (Supplementary Table 1).

**Table 3:** Patients’ and General Practitioners’ Preferences for Deprescribing and Medication Decision-making (*n* = 65 GP-Patient Dyads)

Patients (*n* = 65)	*n* (%)	GPs-patient dyads (*n* = 65)	*n* (%)
Deprescribing		Deprescribing	
Thinking about your current medication list, are there any medications that you would like to stop or reduce ^1^		Would you stop or reduce the dosage of any of the medications this patient is currently taking?	
Yes	25 (38%)	Yes	23 (35%)
No	40 (61%)	No	42 (65%)
Missing	1 (2%)	Missing	0 (0%)
Trust		Trust	
Trust in the GP^2^ (range 5–25), *n* (IQR)	23 (5)	Trust in the patient ^1^	
Missing	8 (12%)	This patient tells me everything	
Abbreviated Wake Forest Trust in Physician scale ^2^		Strongly agree/agree	47 (72%)
Sometimes my GP cares more about what is convenient for them than about my medical needs		Don’t know/disagree/strongly disagree	17 (26%)
Strongly agree/agree	4 (6%)	Missing	1 (2%)
Neutral/disagree/strongly disagree	53 (82%)	Sometimes this patient does not follow my recommendations	
Missing	8 (12%)	Strongly agree/agree	14 (22%)
My GP is extremely thorough and careful		Don’t know/disagree/strongly disagree	50 (77%)
Strongly agree/agree	53 (82%)	Missing	1 (2%)
Neutral/disagree/strongly disagree	6 (9%)	This patient trusts me	
Missing	6 (9%)	Strongly agree/agree	62 (95%)
I completely trust my GP's decision about which medical treatments are best for me		Don’t know/disagree/strongly disagree	3 (4%)
Strongly agree/agree	56 (86%)	Missing	2 (3%)
Neutral/disagree/strongly disagree	6 (9%)	This patient often disagrees with my recommendations	
Missing	3 (5%)	Strongly agree/agree	1 (2%)
My GP is completely honest about the different treatment options available for my health problem		Don’t know/disagree/strongly disagree	63 (96%)
Strongly agree/agree	54 (83%)	Missing	1 (2%)
Neutral/disagree/strongly disagree	6 (9%)		
Missing	5 (8%)		
All in all, I have complete trust in my GP			
Strongly agree/agree	59 (91%)		
Neutral/disagree/strongly disagree	4 (6%)		
Missing	1 (2%)		

^1^Questions were developed by the study team for this survey

^2^Abbreviated Wake Forest Trust in Physician Scale^25^. Score is within 5 to 25, with higher values indicating higher trust

Each patient responded to each question once, while GPs provided a separate response for each of their patients. As a result, the total number of responses was 65 for both groups

Missing is only shown for variables with missing data

Concordance between GPs and patients on deprescribing at least one medication was observed in only 8 dyads, with just one dyad identifying the same medication (opioid analgesic: Tapentadol) (Supplementary Table 1).

**Figure 1:**
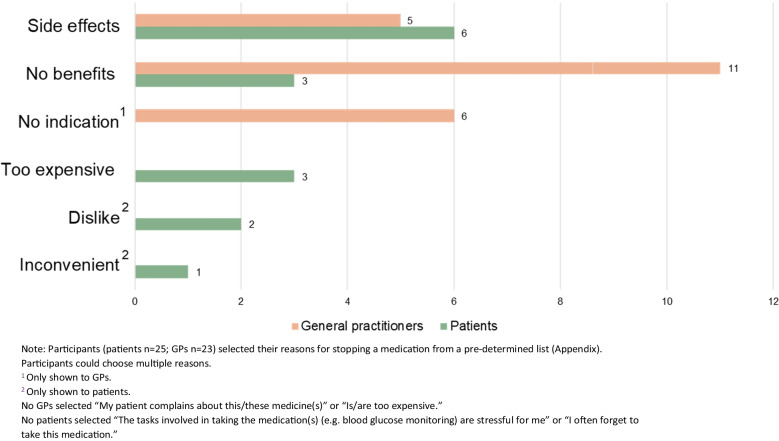
Reasons why general practitioners and patients were interested in deprescribing (absolute numbers).

The three most reported types of medication participants identified for deprescribing were dietary supplements (*GPs* = 11/42, 26%; *patients* = 4/35, 11%), cardiovascular system (*GPs* = 9/42, 21%; *patients* = 15/35, 43%), and the nervous system (*GPs* = 8/42, 19%; *patients* = 9/35, 26%) medications (Supplementary Fig. 1, Supplementary Table 2). Patients reported side effects (9%), lack of benefit (5%), and high cost (5%) as the main reasons for wanting to deprescribe (Fig. 1), whereas GPs most often reported lack of benefit (17%), side effects (9%), and no clinical indication (9%).

**Figure 2:**
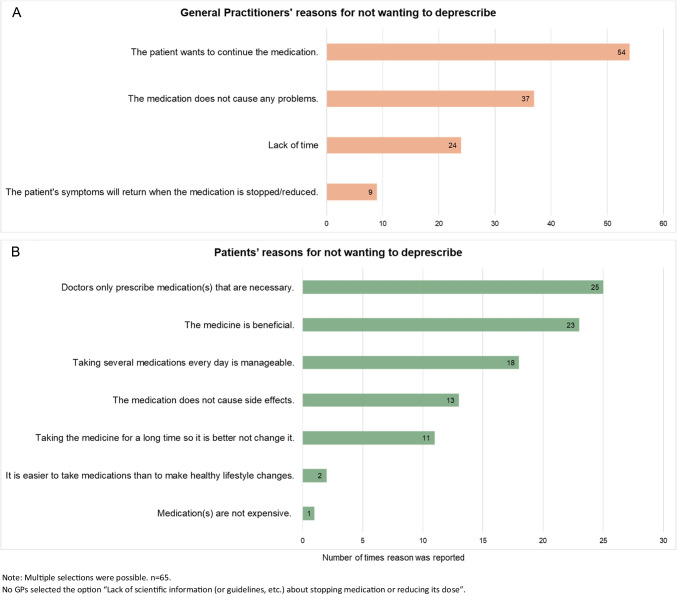
Reasons why general practitioners and patients were not interested in deprescribing (absolute numbers).

The main reason GPs did not want to deprescribe (Fig. 2A) was the belief that the patient wanted to continue the medication (83%, 54/65), followed by the medication did not cause problems (57%, 37/65), and a lack of time (37%, 24/65). Patients reported not wanting to deprescribe (Fig. 2B) because they believe doctors only prescribe necessary medications (38%, 25/65), medications are beneficial (35%, 23/65), and taking multiple medications is manageable (28%, 18/65).

### Decision-making, Goals, and Trust

GPs who were interested in deprescribing and those who were not rated understanding patient goals as very important (50% vs. 50%) and reported talking to their patients about their goals and preferences (Supplementary Table 3). Among the 65 GP-patient dyads, 35% (*n* = 23) of GPs indicated they would deprescribe a medication for their patient, while 65% (*n* = 42) would not (Supplementary Table 4).

Among the 65 patients, 39% (*n* = 25) expressed interest in deprescribing a medication, while 61% (*n* = 39) did not. Patients who wanted to deprescribe a medication more often rated their health as excellent, very good, or good (60% vs. 36%) and reported higher confidence in filling out medical forms (72% vs. 56%). Satisfaction with current medications was high in both groups (88% vs. 97%), and most patients preferred to be informed but trusted their doctor to make medication decisions (92% vs. 82%) (Supplementary Table 5).

Overall, patients reported a high level of trust in their GPs (median score = 23, out of 25). In univariate analyses, GPs who strongly agreed or agreed with the statement “*This patient tells me everything*” had a lower likelihood of wanting to deprescribe (RR = 0.63, 95%CI = 0.46 to 0.90) (Table 4).

**Table 4:** Association Between Trust and Interest in Deprescribing^1^

**Part A. Association between patient-reported trust in their GP (Wake Forest score) and patient interest in deprescribing** (***n*** = 63) ^**2**^		
	Univariate model	
	Relative risk	95% confidence interval
Higher trust in the GP (ref. lower trust)^3^	0.93	0.40 to 2.13
**Part B. Association between GP-reported patient trust and GP interest in deprescribing** (***n*= 64)** ^**4**^		
“This patient tells me everything” ^5^		
Yes	0.63	0.44 to 0.90
No	ref	ref
“Sometimes this patient does not follow my recommendations” ^6^		
Yes	1.02	0.41 to 2.52
No	ref	ref

^1^We used Generalized Estimating Equations (GEE) with a Poisson distribution, log link, and robust standard errors to estimate risk ratios, accounting for within-GP correlations^37^. A Kauermann-Carroll finite-sample correction was applied to the standard errors^40^

^2^Outcome: patient interest in deprescribing, defined as a “yes” to the question “Thinking about your current medication list, are the are any medications you would like to stop or reduce?” (yes: *n* = 25, 38%)

^3^Abbreviated Wake Forest Trust in Physician Scale^25^(range = 5–25); scores ≥ 23 (sample median) indicate higher trust

^4^Outcome: GP interest in deprescribing, defined as “yes” to the question “Would you stop or reduce any medication for this patient?” (yes: *n* = 23, 35%)

^5^Responses of *strongly agree or agree* were categorized as “yes,” while *don’t know*, *disagree*, and *strongly disagree* were categorized as “no”

^6^Responses of *strongly agree or agree* were categorized as “yes,” while *don’t know*, *disagree*, and *strongly disagree* were categorized as “no”

## DISCUSSION

Deprescribing is increasingly recognized as an essential strategy for medication optimization in older adults; yet achieving this remains challenging. As one of the largest studies comparing GP and patient deprescribing preferences, we found that alignment on specific medications to deprescribe was rare. Our findings support broader evidence showing that up to 75% of older adults decline participation in deprescribing trials^41–43^and acceptance rates of deprescribing recommendations vary widely^44^.

In our study, GPs and patients identified the same top three medication categories as candidates for deprescribing: dietary supplements, cardiovascular, and nervous system medications. To our knowledge, only two other studies, both conducted in the Netherlands and published in Dutch, have compared deprescribing preferences between patients and healthcare providers. Van Marum et al.’s study^45^, set in primary care, involved 40 older adults taking seven or more regular medications, pharmacists, and GPs. Frankowski et al.^46^studied 47 adults recruited from a geriatric psychiatry residential ward, psychiatrists, pharmacists, and GPs. In our study, cardiovascular medications were the second most identified category for deprescribing (GPs = 21%; patients = 26%), which aligns with Frankowski’s findings and other research showing acceptability of discontinuing cholesterol-lowering medications^45,47^. However, exceptions emerged within this category. In van Marum’s study, GPs considered blood thinners essential in 75% of cases, with minimal support for discontinuation across patients, GPs, and pharmacists (0–2%). Similarly, in our study, only one patient and no GPs identified antithrombotic agents as a potential target for deprescribing, even though 51 patients were taking these medications. In Frankowski’s study, patients and healthcare professionals often disagreed about which medications to continue or stop. While 40 of 47 patients identified medications they wanted to continue, only 17 specified drugs they wanted to stop. Patients often expressed a preference to rely on their doctor’s expertise for these decisions, a sentiment also observed in our study. Frankowski et al. found patients could spontaneously recall only 37% of their medications. They also found oppositional preferences between patients and healthcare professionals. At the individual level, about 50% of patients and healthcare professionals did not match in their preferences for continuing specific medications, and approximately 75% disagreed on which medications should be deprescribed, mirroring findings from our study. These findings collectively highlight the complex interplay between patient and healthcare provider preferences around continuing and stopping medications, and emphasizes the importance of incorporating both perspectives in the deprescribing process.

Trust is undeniably important to the therapeutic relationship between patients and their doctors^48^, yet its role in deprescribing preferences is complex. Rather than having a straightforward relationship with deprescribing preferences, trust appears to act as a facilitator for both continuing and stopping medications, depending on the context. Like ours, there is inconsistency in the literature as to whether there is statistical significance between patients’ trust in doctors and their attitudes towards deprescribing^49^. Additionally, in the studies where an association is found, there is inconsistency in the direction of the association (i.e., trust is associated with higher^50,51^ or lower^15^ preference for deprescribing). Trust may motivate patients to follow deprescribing recommendations^10^or initiate deprescribing discussions^52^, but it can equally support medication continuation when patients believe their doctor understands their health needs^53^. Some patients trust their doctor to make all medication-related decisions, believing a long relationship ensures the doctor’s understanding of their goals^4,36^. Indeed, physicians’ comprehensive knowledge of their patient and patients’ trust in their physician are strong predictors of medication adherence^54^. Therefore, trust can support starting, continuing, and stopping medications, depending on individual circumstances, suggesting it is not inherently aligned with any singular preference for deprescribing.

## STRENGTHS AND LIMITATIONS

Our study provides an exploration of patients’ and GPs’ views on deprescribing, offering rare insights into their preferences and decision-making dynamics. By examining specific medications rather than general attitudes, we showed how particular medications are preferred for deprescribing, potentially informing more targeted interventions. However, our study has several limitations to be considered.

While we examined deprescribing preferences for specific medications from the patients’ and GPs’ perspectives, these were reported outside of a clinical consultation. This means that, like much of the existing literature, responses may reflect hypothetical willingness rather than actual deprescribing behaviors. Patients and GPs may be more open to deprescribing when completing a questionnaire rather than when faced with the complexity of a real-life clinical decision. Participants were not informed of each other’s preferences and we do not know if GPs or patients initiated medication changes following the survey. Additionally, the absence of diagnostic and health information meant we were unable to evaluate the appropriateness of the medications identified for deprescribing. We did not assess patients’ decision-making preferences using the Control Preferences Scale^35^ as it overlapped with another item in our survey. Despite instructing GPs to recruit patients consecutively, selection bias cannot be ruled out. Due to the recruitment approach, GPs may have recruited patients more open to medication changes or patients who have higher trust in their GP. Due to an oversight in the survey design, we only collected information on whether patients were aged 65 years or older, limiting our ability to include patient age in our analyses. Our study was conducted in the German-speaking part of Switzerland, so the findings may need to be confirmed in other populations with different healthcare practices. Finally, we recruited fewer GPs than anticipated, limiting both the statistical power to detect patterns in GP-patient deprescribing agreement and the generalizability of the findings. As such, our results should be interpreted as exploratory.

## CONCLUSION

In this study, GPs and patients seldom identified the same medications they would be interested in deprescribing. Our findings suggest that successful deprescribing initiatives need explicit discussions about medication necessity and consideration of both GP and patient perspectives. Future research should explore why explicit deprescribing discussions may not be occurring regularly in clinical practice, whether due to time constraints, clinical priorities, or reliance on assumptions about patient preferences. Interventions that promote open dialogue about medication preferences and address mutual assumptions between patients and GPs may facilitate more effective deprescribing conversations.

## Supplementary information

Below is the link to the electronic supplementary material.

ESM 1 (DOCX 138 KB)

## Data Availability

The data for this study are available to other researchers on request. The data will be made available for scientific research purposes, after the proposed analysis plan has been approved by the core study team. Data and documentation will be made available through a secure file exchange platform after approval of the proposal. In addition, a data transfer agreement must be signed (which defines obligations that the data requester must adhere to regarding privacy and data handling). For data access, please contact the corresponding author.
