# Supplementary material for: Deprescribing Decisions in Swiss Primary Care: Low Concordance Between General Practitioners and Older Adults
Source: J Gen Intern Med. 2026 Jan 14;41(10):2796–806. doi: 10.1007/s11606-025-10081-z (PMC13421698; doi:10.1007/s11606-025-10081-z)
Supplement: Supplementary file 1 — (DOCX 138 KB) [file 11606_2025_10081_MOESM1_ESM.docx]

**Supplementary Material**

**Deprescribing decisions in Swiss primary care: low concordance between general practitioners and older adults**

**Authors**

Kristie Rebecca Weir^1,2^* PhD, ORCID: <https://orcid.org/0000-0002-9507-5050>

Renata Vidonscky Lüthold^1,3^* PhD, ORCID: <https://orcid.org/0000-0001-7475-8648>

Zsofia Rozsnyai^1^ MD

Sven Streit^1^ PhD, MD

Katharina Tabea Jungo^1,4^ PhD, ORCID: <https://orcid.org/0000-0002-1782-1345>

**Share first authorship*

^1^ Institute of Primary Health Care (BIHAM), University of Bern, 3012 Bern, Switzerland

^2^Sydney School of Public Health, Faculty of Medicine and Health, University of Sydney, 2006 Sydney, Australia

^3^ Graduate School for Health Sciences, University of Bern, 3012 Bern, Switzerland

^4^Division of Pharmacoepidemiology and Pharmacoeconomics and Center for Healthcare Delivery Sciences (C4HDS), Department of Medicine, Brigham and Women's Hospital and Harvard Medical School, 02115 Boston, MA, United States

**Table of content**

[**Supplementary Figure 1**. Frequency of the top five medication types identified for deprescribing by general practitioners and patients (n=34 unique medications). 3](#_Toc208329765)

[**Supplementary Table 1.** Concordance between GPs and patients (n=65 GP-patient dyads). 3](#_Toc208329766)

[**Supplementary Table 2.** Medication classes identified by patients and general practitioners (GPs) for deprescribing. 4](#_Toc208329767)

[**Supplementary Table 3.** General practitioner (GP) characteristics and GP-reported preferences towards decision-making and deprescribing, by interest in deprescribing (n=10 GPs) 5](#_Toc208329768)

[**Supplementary Table 4.** GP-reported trust, by interest in deprescribing (n=65 GP-patient dyads) 6](#_Toc208329769)

[**Supplementary Table 5.** Patient characteristics and patient-reported trust by interest in deprescribing (n=65) 7](#_Toc208329770)

[**Survey for patients** 9](#_Toc208329771)

[**Survey for GPs** 14](#_Toc208329772)

# **Supplementary Figure 1**. Frequency of the top five medication types identified for deprescribing by general practitioners and patients (n=34 unique medications).


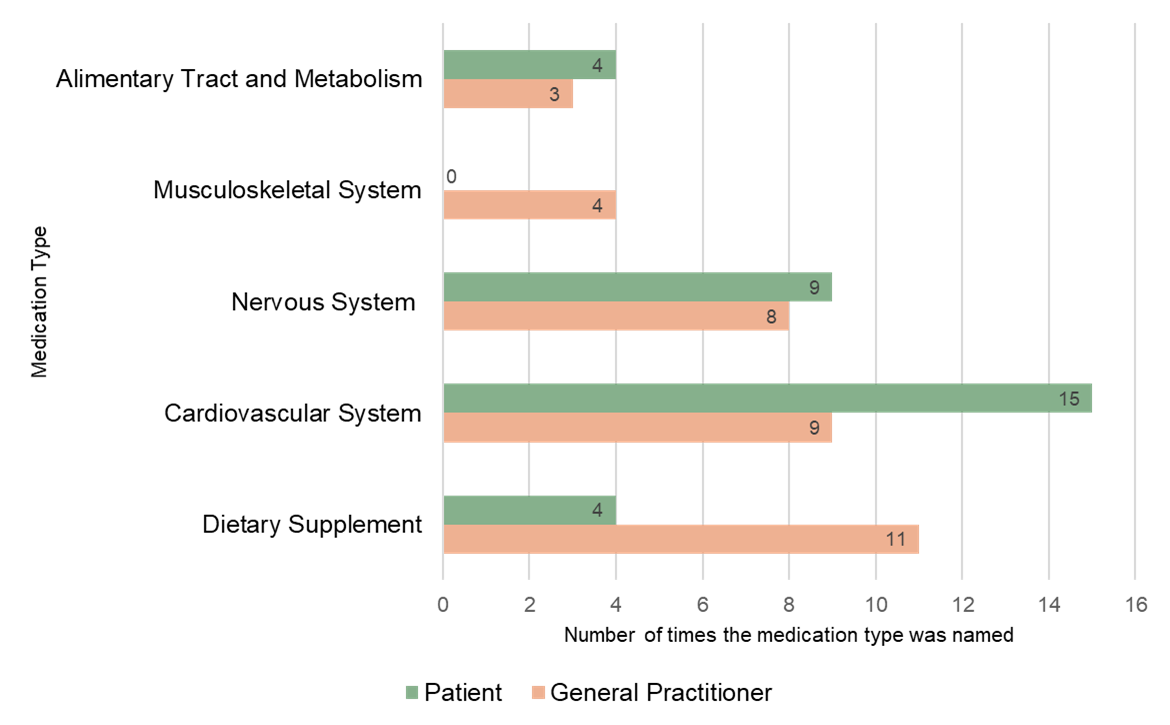


Among the top five medication types identified for deprescribing, GPs identified 42 medications for deprescribing, while patients identified 35.

Note: ATC codes were used to classify medications at the first anatomical level to create therapeutic and pharmacological subcategories.

| **Supplementary Table 1.** Concordance between GPs and patients interest in deprescribing (n=65 GP-patient dyads). | | | |
| --- | --- | --- | --- |
|  |  | **GPs’ interest in deprescribing** | |
|  |  | Yes | No |
| **Patients’ interest in deprescribing**^a^ | Yes | 8 (12%) | 17 (26%) |
|  | No | 15 (23%) | 24 (37%) |

^a^ Missing = 1

# **Supplementary Table 2.** Medication classes identified by patients and general practitioners (GPs) for deprescribing.

| **Medication** | **Patients (n=35 medications) ^b^** | **GPs (n=42 medications) ^c^** |
| --- | --- | --- |
| **Alimentary tract and metabolism ^a^** |  |  |
| Drugs for acid-related disorders | 2 (6%) | 2 (5%) |
| Drugs used in diabetes | 2 (6%) | 1 (2%) |
| **Blood and blood-forming organs** |  |  |
| Antithrombotic agents | 1 (3%) | 0 (0%) |
| **Cardiovascular system ^a^** |  |  |
| Cardiac therapy | 0 (0%) | 2 (5%) |
| Diuretics | 3 (9%) | 2 (5%) |
| Calcium channel blockers | 2 (6%) | 0 (0%) |
| Agents acting on the renin-angiotensin system | 6 (17%) | 1 (2%) |
| Lipid-modifying agents | 4 (11%) | 4 (10%) |
| **Genito-urinary system and sex hormones** |  |  |
| Urologicals | 2 (6%) | 1 (2%) |
| **Systemic hormonal preparations** |  |  |
| Thyroid therapy | 0 (0%) | 2 (5%) |
| Corticosteroids for systemic use | 0 (0%) | 1 (2%) |
| **Antineoplastic and immunomodulating agents** |  |  |
| Antineoplastic agents | 0 (0%) | 1 (2%) |
| **Musculoskeletal system ^a^** |  |  |
| Anti-inflammatory and antirheumatic products | 0 (0%) | 2 (5%) |
| Topical products for joint and muscular pain | 0 (0%) | 1 (2%) |
| Anti-gout preparations | 0 (0%) | 1 (2%) |
| **Nervous system ^a^** |  |  |
| Analgesics | 5 (14%) | 3 (7%) |
| Antiparkinson drugs | 0 (0%) | 1 (2%) |
| Psycholeptics | 1 (3%) | 3 (7%) |
| Psychoanaleptics | 3 (9%) | 1 (2%) |
| **Respiratory system** |  |  |
| Cough and cold preparations | 0 (0%) | 1 (2%) |
| **Other** | 0 (0%) | 1 (2%) |
| **Dietary supplements** ^a^ | 4 (11%) | 11 (26%) |

Note: ATC codes were used to classify medications at the first anatomical level (ATC level 1) and second therapeutic groups (ATC level 2). It was difficult to classify dietary supplements with ATC codes, therefore they are classified as a separate category.

^a^ Corresponds to one of the top five most common medication groups (ATC level 1) identified for deprescribing by GPs and patients.

^b^ Patients: n=25.

^c^ GPs: 10 GPs reported medications for 23 patients of their enrolled patients.

# **Supplementary Table 3.** General practitioner (GP) characteristics and GP-reported preferences towards decision-making and deprescribing, by interest in deprescribing(n=10 GPs)

|  | ***GPs interest in deprescribing at least one medication for any of their enrolled patients*** | |
| --- | --- | --- |
|  | Yes  (n=8, 80%) | No  (n=2, 20%) |
| **GP gender** |  |  |
| Female | 2 (25%) | 1 (50%) |
| Male | 6 (75%) | 1 (50%) |
| ***How often do you talk to your patients about their goals and preferences?*** | | |
| *Sometimes* | 2 (25%) | 0 (0%) |
| *Often* | 6 (75%) | 1 (50%) |
| *Always* | 0 (0%) | 1 (50%) |
| ***How important do you think it is to understand your patients' goals and preferences regarding their medications?*** | | |
| *Quite important* | 4 (50%) | 1 (50%) |
| *Very important* | 4 (50%) | 1 (50%) |
| ***How do you usually make decisions about stopping a medication or reducing its dose with a patient during your consultation?*** | | |
| *The patient makes the final decision about stopping or reducing the dose of a medication.* | 0 (0%) | 1 (50%) |
| *The patient makes the final decision about stopping a medication or reducing its dose after seriously considering my opinion.* | 2 (25%) | 1 (50%) |
| *The patient and I share the responsibility of deciding which medication is best for them.* | 5 (63%) | 0 (0%) |
| *I make the final decision about stopping a medication or reducing its dose, but seriously consider the patient's opinion.* | 1 (13%) | 0 (0%) |
| *I make the final decision about stopping a medication or reducing its dose.* | 0 (0%) | 0 (0%) |
| ***How would you like to make decisions about stopping a medication or reducing its dose with a patient during your consultation?*** | | |
| *The patient makes the final decision about stopping or reducing the dose of a medication.* | 0 (0%) | 1 (50%) |
| *The patient makes the final decision about stopping a medication or reducing its dose after seriously considering my opinion.* | 1 (13%) | 1 (50%) |
| *The patient and I share the responsibility of deciding which medication is best for them.* | 5 (63%) | 0 (0%) |
| *I make the final decision about stopping a medication or reducing its dose, but seriously consider the patient's opinion.* | 1 (13%) | 0 (0%) |
| *I make the final decision about stopping a medication or reducing its dose.* | 1 (13%) | 0 (0%) |
| ***For patients taking medications that could potentially be stopped or reduced: What percentage of them have you recommended this to?*** | | |
| *<70%* | 2 (25%) | 1 (50%) |
| *≥70%* | 6 (75%) | 1 (50%) |

# **Supplementary Table 4.** GP-reported trust, by interest in deprescribing (n=65 GP-patient dyads)

|  | ***Would you stop or reduce any medication for this patient?*** | |
| --- | --- | --- |
|  | Yes  (n=23, 35%) | No  (n=42, 65%) |
| ***This patient tells me everything*** |  |  |
| *Strongly Agree/Agree* | 15 (65%) | 32 (78%) |
| *Don’t know/Disagree/Strongly Disagree* | 8 (35%) | 9 (22%) |
| ***Sometimes this patient does not follow my recommendations*** |  |  |
| *Strongly Agree/Agree* | 5 (22%) | 9 (22%) |
| *Don’t know/Disagree/Strongly Disagree* | 18 (78%) | 32 (78%) |

# **Supplementary Table 5.** Patient characteristics and patient-reported trust by interest in deprescribing (n=65)

|  | ***Thinking about your current medication list, are there any medications that you would like to stop or reduce?* ^1^** | |
| --- | --- | --- |
|  | Yes (n=25, 39%) | No (n=39, 61%) |
| **Patient gender** |  |  |
| Female | 12 (48%) | 16 (41%) |
| Male | 13 (52%) | 22 (56%) |
| Missing | 0 | 1 (3%) |
| **Trust in the General Practitioner (GP)^2^** |  |  |
| Higher trust | 14 (56%) | 24 (62%) |
| Lower trust | 11 (44%) | 14 (36%) |
| Missing | 0 | 1 (3%) |
| ***What is your highest completed education?*** |  |  |
| *None/primary school* | 9 (36%) | 13 (33%) |
| *Secondary school/third level education* | 16 (64%) | 25 (64%) |
| Missing | 0 | 1 (3%) |
| **Self-rated health** |  |  |
| *Excellent/very good/good* | 15 (60%) | 14 (36%) |
| *Average/poor* | 10 (40%) | 23 (59%) |
| Missing | 0 | 2 (5%) |
| ***How confident are you filling out medical forms by yourself?*** |  |  |
| *Extremely/quite a bit* | 18 (72%) | 22 (56%) |
| *Somewhat/a little bit/not at all* | 7 (28%) | 17 (44%) |
| ***How long have you been seeing this GP?*** |  |  |
| *< 19 years* | 19 (76%) | 29 (74%) |
| *20 years +* | 6 (24%) | 8 (21%) |
| Missing | 0 | 2 (5%) |
| ***Overall, I am satisfied with my current medications*** |  |  |
| *Strongly agree/agree* | 22 (88%) | 38 (97%) |
| *Unsure/disagree/strongly disagree* | 3 (12%) | 1 (3%) |
| ***How do you make decisions about your medications?*** | | |
| *I want to be informed, but I trust my doctor to make decisions about my medications.* | 23 (92%) | 32 (82%) |
| *I make decisions about the medications I take, or share the decision with my doctor.* | 2 (8%) | 0 (0%) |
| *Other people (e.g. my doctor or my partner) make decisions for me about my medications.* | 0 (0%) | 0 (0%) |
| Missing | 0 | 7 (18%) |
| ***How do you get information about your medications?*** | | |
| *My doctor and I talk about my medications together.* | 14 (56%) | 26 (67%) |
| *I know about my medications – I ask my doctor or read the information leaflet or search online.* | 9 (36%) | 6 (15%) |
| *I don't know much about my medications.* | 1 (4%) | 1 (3%) |
| Missing | 1 (4%) | 6 (15%) |
| ***What do you think about the medications you are taking?*** | | |
| *My medications are important, they keep me alive and help me live well.* | 10 (40%) | 18 (46%) |
| *My medications are doing what they're supposed to do.* | 11 (44%) | 9 (23%) |
| *I don't worry too much about my medication; I take what my doctor prescribes.* | 4 (16%) | 6 (15%) |
| *Missing* | 0 (0%) | 6 (15%) |

^1^ Missing = 1 (2%).

^2^ Trust in the General Practitioner (GP) using the abbreviate Wake Forest score.^19^ Scores higher than the median (≥22) were considered high trust in the GP. Percentages were regarding the total number of responses in each group.

# **Survey for patients**

Have you already signed the consent form together with your GP?

*If you click/check "Yes", you agree to participate.*

- Yes (automatic forwarding to the questionnaire)
- No (end of study participation)

**Questions about the inclusion criteria**

1. How old are you (in years)?

- 65 years old or older (continue to the next question)
- 64 years old or younger (end of study participation)

1. Do you regularly take 5 or more medications? (Regularly means: every day or most days for 30 days or more)

- Yes (continue to the next question)
- No (end of study participation)

1. Do you live in Switzerland?

- Yes (go to next question)
- No (end of study participation)

**Information about your GP**

1. What is the name of your general practitioner? Please enter their first and last names: ___________________
2. What is the location of your GPs’ office? _________________________________________________________
3. What is the postcode of the practice?___________________________________________________________
4. What is the name of the street where the GP practice is located? _____________________________________
5. Is this your family doctor? (Definition: if you have a health problem, you usually always consult the same family doctor - except in emergencies

- Yes (continue with the next question)
- No (Please go directly to Section Socio-demographic questions)

1. If yes, how long have you been going to this family doctor?

- 0-9 years
- 10-19 years
- 20-29 years
- 30+ years

**Socio-demographic questions**

We will now ask you some questions in order to better understand who answered our questionnaire.

1. What is your gender?

- Male
- Female
- Other

1. What area do you live in?

- Urban
- Suburban
- Rural

1. What is your postcode? _____________
2. Do you live alone in your household?

- Yes
- No

1. What is your living situation?

- Own your house or apartment
- Rented house or apartment

1. What is your highest completed education?

- None
- Primary school
- Secondary education (apprenticeship or high school)
- Tertiary education (university or college studies)

1. How do you make ends meet financially?

- With great difficulty
- With some difficulty
- Quite easily
- Without any problems

1. Where were you born?

- In the country where I currently live
- Other country [Please specify country] ______________________

1. What is your first language?

- Official language of the country where I currently live
- Other language [Please specify language] ___________________

1. How confident are you in filling out medical forms by yourself?

- Not at all
- A little bit
- Somewhat
- Quite a bit
- Extremely

1. Generally speaking, how would you describe your health today?

- Excellent
- Very good
- Good
- Average
- Poor

**Questions about your use of medication**

Now we would like to learn more about your experiences with taking medication.

1. I prepare my medication myself:

- Yes, I prepare and take it myself according to the prescription.
- No, I receive support in preparing/taking my medication from relatives, the Spitex or at the pharmacy for example.

1. Overall, I am satisfied with my current medications.

- Strongly agree
- Agree
- Unsure
- Disagree
- Strongly disagree

1. How many different kinds of medications do you take regularly? (Regularly means daily or on most days of the week.) Please indicate the number of different kinds of medications.

Number of different medications: ___________________________________

**Questions about your attitude towards and decisions about medication**

Now we are going to ask you questions on your thoughts about stopping or reducing the dose of medicines.

1. Thinking about your current medication list, are there any medications that you would like to stop taking or reduce the dose of?

- Yes (please continue to the next question)
- No, I am not considering stopping or reducing the dose of any medication. (Please go to Question 26)

1. In the following table, please state the name(s) of the medication(s) that you would consider stopping or reducing, and the reason why.

*Any lines that are not applicable can be left empty.*

| Name(s) of the medication(s) that you would consider stopping or reducing | Why did you choose this/these medication(s) to stop or reduce?  *Please check all answers that apply* |
| --- | --- |
| Name of the medication:      __________________________ | - It causes side effects. - I do not benefit from it. - I do not like the medication. - The medication is too expensive. - It is inconvenient for me to take this medication. - The tasks involved in taking the medication(s) (e.g. blood glucose monitoring) are stressful for me. - I often forget to take this medication. - Other reason:___________________________ |
| Name of the medication:    __________________________ | - It causes side effects. - I do not benefit from it. - I do not like the medication. - The medication is too expensive. - It is inconvenient for me to take this medication. - The tasks involved in taking the medication(s) (e.g. blood glucose monitoring) are stressful for me. - I often forget to take this medication. - Other reason:___________________________ |
| Name of the medication:      __________________________ | - It causes side effects. - I do not benefit from it. - I do not like the medication. - The medication is too expensive. - It is inconvenient for me to take this medication. - The tasks involved in taking the medication(s) (e.g. blood glucose monitoring) are stressful for me. - I often forget to take this medication. - Other reason:___________________________ |
| Name of the medication:      __________________________ | - It causes side effects. - I do not benefit from it. - I do not like the medication. - The medication is too expensive. - It is inconvenient for me to take this medication. - The tasks involved in taking the medication(s) (e.g. blood glucose monitoring) are stressful for me. - I often forget to take this medication. - Other reason:___________________________ |

*After the table please continue to section “additional questions about stopping medications and your willingness to do so”.*

1. You may not want to stop taking a medication or reduce the dose. Here are some reasons why. Which one(s) do you think are the most important reasons for not stopping a medication? (Please select all that apply)

- The medicine is beneficial.
- Taking the medicine for a long time so it is better not change it.
- Taking several medications every day is manageable.
- The medication does not cause side effects.
- Medication(s) are not expensive.
- Doctors only prescribe medication(s) that are necessary.
- It is easier to take medications than to make healthy lifestyle changes.
  - Other reasons: _____________________________________________

**Additional questions about stopping medication and your willingness to do so:**

For each of the following, please select the statement that best aligns with your views.

1. What do you think about the medications you take?

- My medications are important, they keep me alive and help me live well.
- My medications do what they are supposed to do.
- I don’t really care much about my medications, I take them as my doctor tells me to.

1. How do you get information about your medications?

- My doctor and I talk about my medications together.
- I know about my medications – I ask my doctor or read the information leaflet or search online.
- I don't know much about my medications.

1. How do you make decisions about your medications?

- I want to be informed, but I trust my doctor to make decisions about my medications.
- I make decisions about the medications I take, or share the decision with my doctor.
- Other people (e.g. my doctor or my partner) make decisions for me about my medications.

1. What do you think about the idea of stopping or reducing the dose of one or more of your medications?

- I would not like to stop any of my medications or reduce the dose.
- I wish I did not take so many medications and I would stop or reduce the dose of my medications if I could.
- If my doctor said that it is possible to stop or reduce the dose of a medication that would be ok with me.

**Questions about your relationship to your family doctor**

This section is about your relationship with your GP and your trust in them. Please indicate how strongly you agree with each of the statements. There are no right or wrong answers.

|  | Strongly disagree | Disagree | Neutral | Agree | Strongly agree |
| --- | --- | --- | --- | --- | --- |
| 1. Sometimes my GP cares more about what is convenient for them than about my medical needs. |  |  |  |  |  |
| 1. My GP is extremely thorough and careful. |  |  |  |  |  |
| 1. I completely trust my GP's decision about which medical treatments are best for me. |  |  |  |  |  |
| 1. My GP is completely honest about the different treatment options available for my health problem. |  |  |  |  |  |
| 1. All in all, I have complete trust in my GP. |  |  |  |  |  |

**Final questions**

1. Did anyone help you with completing this questionnaire?

- No
- Yes
  - If yes: Who? (please check the answer that applies)
    - Relatives
    - Friends
    - GP
    - GP practice staff
    - Other: _____________________________________________________

You had the opportunity to complete the questionnaire online or on paper.

- “I confirm that I only completed one of the versions of the questionnaire.”

Thank you for your participation, you can now close the survey. If you completed the questionnaire online, you can now close the window. If you completed the questionnaire on paper, please return it to your GP practice as soon as possible.

# **Survey for GPs**

**Part 1. GP Profile**

*Please complete once as part of this study.*

| Questions about yourself | |
| --- | --- |
| 1. Full name | ______________________________________________ |
| 2. Address of the practice where you work | ______________________________________________ |
| 3. Town and postcode of the practice where you work | ______________________________________________ |
| 4. Location of the practice where you work | *Please check the most appropriate answer.*   - Urban - Suburban - Rural |
| 5. Please indicate your gender | - Male - Female - No answer |
| 6. Please indicate your age: | - ______________________________________________ |
| 7. What is your first language? | - German/Swiss German - French - Italian - Other: _____________________________________ |
| 8. Do you have an FMH title? | - Yes - No |
| If yes: Which FMH title do you have? | ______________________________________________ |
| Questions about your daily work | |
| 9. How much experience do you have as a general practitioner? (in years) | ______________________________________________ |
| 10. On how many half-days per week do you see patients? (one half-day equals 10%) | ______________________________________________  (please give a number between 1-10) |
| 11. How many consultations do you have on an average workday (this corresponds to two half-days)? | - <15 - 15-25 - 26-35 - >35 |
| 12. What kind of practice do you work in? | - Single practice - Group practice |
| If group practice: How many GPs work in this practice? | ______________________________________________ |
| 13. Before you were invited to participate in this project: Had you ever heard of the concept of deprescribing? | - Yes - No |
| General questions about your patients with polypharmacy | |
| 14. Please estimate the percentage of patients in your practice who have polypharmacy (i.e. who regularly take 5 or more medications)? | ______________________________________________  (Please enter a number between 0-100) |
| 15. Please estimate the percentage of patients in your practice who are eligible for stopping or dose reduction? | ______________________________________________  (Please enter a number between 0-100) |
| 16. For patients taking medications that could potentially be stopped or reduced: What percentage of them have you recommended this to? | ______________________________________________  (Please enter a number between 0-100) |
| 17. If you did not recommend stopping or reducing the dose of medication, what were the main reasons? | ***Please check all answers that apply.***   - Lack of time - The medication does not cause any problems. - The patient wants to continue the medication. - The patient's symptoms will return when the medication is stopped/reduced. - Lack of scientific information (or guidelines, etc.) about stopping medication or reducing its dose - Other reason: __________________________________ |
| Questions about decision-making | |
| 18. How important do you think it is to understand your patients' goals and preferences regarding their medications? | - Not at all important - A little important - Somewhat important - Quite important - Very important |
| 19. How often do you talk to your patients about their goals and preferences? | - Never - Rarely - Sometimes - Often - Always |
| 20. Please select the option that best reflects how you usually make decisions about stopping a medication or reducing its dose with a patient during your consultation. | - The patient makes the final decision about stopping or reducing the dose of a medication. - The patient makes the final decision about stopping a medication or reducing its dose after seriously considering my opinion. - The patient and I share the responsibility of deciding which medication is best for them. - I make the final decision about stopping a medication or reducing its dose, but seriously consider the patient's opinion. - I make the final decision about stopping a medication or reducing its dose. |
| 21. Please select the option that best describes how you would like to make decisions about stopping a medication or reducing its dose with a patient in your consultation. | - The patient makes the final decision about stopping or reducing the dose of a medication. - The patient makes the final decision about stopping a medication or reducing its dose after seriously considering my opinion. - The patient and I share the responsibility of deciding which medication is best for them. - I make the final decision about stopping a medication or reducing its dose, but seriously consider the patient's opinion. - I make the final decision about stopping a medication or reducing its dose. |

You had the opportunity to choose between filling out an online or a paper questionnaire.

o “I confirm that I have only completed one version of the two questionnaires”

Thank you very much for completing this questionnaire. We appreciate you taking the time to do so.

**Part 2. Questions about the hypothetical discontinuation of medications or reduction of their dose in the patients recruited by you**

Please complete one form per patient recruited for this study.

**Procedure:**

1) After you have recruited 5 patients for this study, have their current medication list (digital or on paper) at hand.

2) Then fill out this short questionnaire for all 5 patients and send us their current medication lists (with your comments). You can do this by e-mail or by post

| **Questions about you** | | | | | | |
| --- | --- | --- | --- | --- | --- | --- |
| 1. Full name | ___________________________________________  *We need this information in order to be able to assign the participating patients to the participating GPs.* | | | | | |
| **Questions about the Patient** | | | | | | |
| 2. Patient’s full name | ________________________________________ | | | | | |
| 3. Patient’s address | _________________________________________ | | | | | |
| 4. How long has this patient been your patient? | - 0-9 years - 10-9 years - 20-29 years - 30+ years | | | | | |
| **Questions about the patient's use of medication** | | | | | | |
| 5. How many long-term medications (prescribed for ≥30 days) are currently prescribed for this patient? | ______________________________________  Please enter a number. | | | | | |
| 6. Which long-term medications (prescribed for ≥30 days) are currently prescribed for this patient? | Please take the list of medications you have for this patient.  **Mark an X** for all long-term medications (prescribed for ≥30 days).  Example: **X** Pantoprazole 20mg, 1x per day | | | | | |
| 7. Would you stop or reduce the dose of any of the medications that the patient is currently taking? | - Yes - No | | | | | |
| 8. If you were to think about stopping or reducing the dose of one of the medicines this patient is currently taking, which would it be? | Please **mark** these medicines with a **circle**.  Example: Pantoprazole 20mg, 1x per day **O** | | | | | |
| 9. Please indicate why you have chosen this/these medication(s) to discontinue or reduce their dose: | Mark all the answers that apply: The medication(s)...   - Has/have side effects - Has/have no benefit - Has/have no indication - Is/are too expensive - My patient complains about this/these medicine(s) - Other reason:______________________________ | | | | | |
| Now please go through the statements below and indicate to what extent you agree with them. | | | | | | |
|  | Strongly disagree | Disagree | Don’t know | | Agree | Strongly agree |
| 10. This patient tells me everything. |  |  |  | |  |  |
| 11. Sometimes this patient does not follow my recommendations. |  |  |  | |  |  |
| 12. This patient trusts me. |  |  |  | |  |  |
| 13. This patient often disagrees with my recommendations. |  |  |  | |  |  |
|  |  |  |  |  |  |  |

You had the opportunity to choose between filling out an online or a paper questionnaire.

o “I confirm that I have only completed one version of the two questionnaires”

Thank you for completing this questionnaire. We appreciate you taking the time to do this.
